# Half of annotated human microRNAs are expressed at levels of questionable biological significance

**DOI:** 10.1016/j.isci.2025.113445

**Published:** 2025-08-25

**Authors:** Saba Ataei Kachooei, Julie M. Bracken, Katherine A. Pillman, Philip A. Gregory, Cameron P. Bracken

**Affiliations:** 1 Centre for Cancer Biology, An Alliance of SA Pathology and University of South Australia, Adelaide, SA, Australia; 2 School of Medicine, Discipline of Medicine, University of Adelaide, Adelaide, SA, Australia; 3 School of Biological Sciences, Faculty of Sciences, University of Adelaide, Adelaide, SA, Australia

**Keywords:** Molecular mechanism of gene regulation, Cell biology, Systems biology

## Abstract

MicroRNAs (miRNAs) are widely studied for their role in post-transcriptional gene regulation, often using exogenous overexpression to infer function. However, such approaches may not reflect physiological activity, as they rely on unnaturally high expression levels. To address this, we determined the minimal threshold required for detectable endogenous miRNA function using sensitive reporters. Comparison with small RNA sequencing data from thousands of cell lines and tissues revealed that over half of all miRNAs never reach functionally relevant levels, with more recently evolved miRNAs generally exhibiting lower expression. This challenges the validity of numerous studies reporting roles for lowly expressed miRNAs, suggesting many findings reflect artifacts of overexpression rather than true biological activity. Our work underscores the importance of evaluating miRNA function in a native context and supports the view that the functional human “microRNAome” is substantially smaller than estimates based solely on sequencing data.

## Highlights

• Exogenous miRNA expression does not reflect endogenous function
• Establishment of a minimal level of expression for detectable miRNA function
• Half of miRNAs are expressed at insufficient levels for detectable function
• More recently evolved miRNAs tend to be expressed at lower levels

## Graphical abstract

**Figure:**
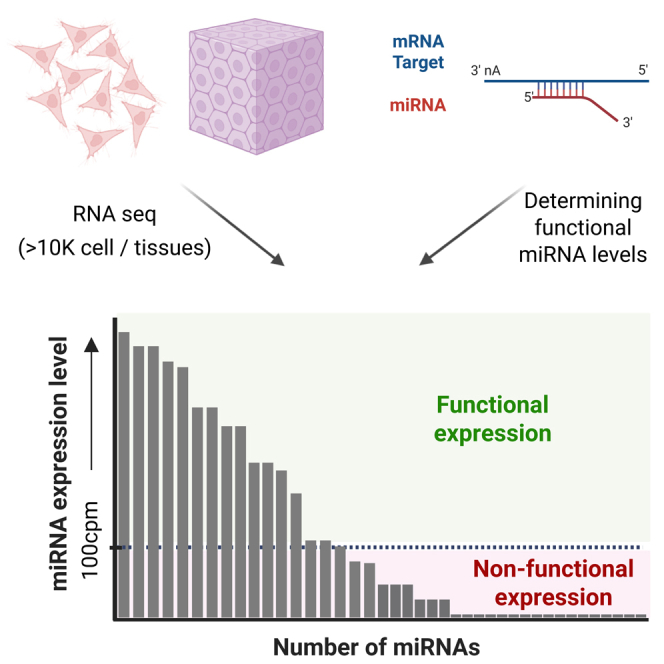

## Introduction

MicroRNAs (miRNAs) act as the target recognition component of a larger ribonucleoprotein complex called RISC (RNA-induced silencing complex), where, in a sequence specific manner, they act to bring target mRNAs to RISC via complementary base pairing. RISC then suppresses the target mRNA through a combination of mRNA destabilization and translational repression.^1^^,^^2^ Initially discovered in *Caenorhabditis elegans in 1993*^3^^,^^4^, interest in the field rapidly grew with their subsequent discovery in other branches of life; initially flies then both mammals and plants. Mechanisms of miRNA biogenesis and function were then elucidated, with each newly discovered miRNA annotated and placed into databases from which miRbase emerged as the primary resource.^5^

Perhaps surprisingly, however, given the miRNA field spans ∼200k publications, the number of genuine human miRNAs has remained contentious. Some estimates derived from the analysis of extensive sequencing data has placed the number of human miRNAs at over 5000,^6^ whilst others propose < 600.^7^^,^^8^ The disagreement lies with separating genuine, functional miRNAs from the RNA degradome, and to what extent non-canonical features should be used as exclusionary factors. For example, to what degree should one require high 5′-homogeneity, high “in-cluster ratio” (the proportion of local reads mapping to a specific miRNA), and the presence of sequences mapping to the other arm of a hairpin structure from which miRNAs are canonically produced^8^? These questions are especially difficult given that there are non-canonical pathways that also give rise to functional miRNAs.^9^^,^^10^^,^^11^^,^^12^^,^^13^^,^^14^^,^^15^

Beyond the question of what features of small RNAs should be regarded as exclusionary, there is also a fundamental question of how much miRNA needs to be present to be biologically meaningful? miRNA activity is dose-dependent, but since the use of miRNA mimics leads to supra-physiological levels of expression and the displacement of endogenous miRNAs from AGO, their use may not be representative of authentic miRNA function.^16^ Further, if a contentiously annotated miRNA is expressed at levels insufficient to be biologically meaningful, the status of the RNA itself is moot.

To address this, we sought to investigate the minimal level of endogenous expression that is required to warrant the consideration of miRNA function. To do so, highly sensitive reporters were constructed for selected miRNAs. By cross-referencing reporter activity against endogenous miRNA expression as determined by RNA-seq, one can estimate the minimal amount of miRNA that needs to be present for detectable activity. In general agreement with previous work,^17^^,^^18^ even when we use reporters that are fully complementary to the miRNA and thus, should be far more efficiently repressed than endogenous targets, we note miRNA activity is never detectable at less than 200 counts per million (cpm, as determined by small RNA-seq). At levels greater than 1000 cpm the activity of most miRNAs are discernible, though higher expression does not always correlate with stronger repressive activity.

We then use this information to interrogate small RNA sequencing data and find that from 2,817 annotated miRNAs, more than 1,500 are never expressed above 100 cpm in any of the hundreds of cell lines or thousands of tissues examined. These datasets are extensive. In one, 333 diverse cell lines and 10,740 normal and pathologic tissue samples are present.^19^ In another, 196 distinct tissue types have been sequenced across >2000 sequencing runs.^20^ Whilst it is always possible there exist specific cells or developmental contexts that are not captured in this data, this work suggests skepticism should be applied to the biological significance of hundreds of annotated miRNAs simply based upon their expression level. This is in addition to further questions regarding the validity of miRNAs based upon mis-annotation, imprecise processing and inefficient AGO binding.^7^^,^^8^^,^^21^^,^^22^^,^^23^ We further show that the majority of annotated miRNAs that are not listed in miRGeneDB,^7^^,^^21^ and thus whose significance is already questionable on account of their imprecise processing, are also lowly expressed. Almost all miRNAs encoded by loci of ancient taxonomic lineage are highly expressed, whereas approximately 20% of newly emerged miRNAs (human-specific) fail to meet an expression threshold at which reporter gene suppression is detectable. Despite their questionable biological significance, lowly expressed miRNAs are the collective subject of >2,600 articles featuring the name of the miRNA in the title, and >6,500 articles where the miRNA features in the abstract.

Whilst the problem of non-physiological miRNA expression has been described,^24^ and an effort previously made to establish functional miRNA levels,^18^ to our knowledge, this is the first study to cross-reference the level of miRNA expression required for discernible function with the actual expression of miRNAs across the big datasets that are now available. Our findings highlight the importance of considering endogenous miRNA expression, and call into question the findings of thousands of studies whose conclusions we expect are solely attributable to supraphysiological expression. As such, this work should be of value both to researchers before embarking on new miRNA-related projects, and to peer reviewers evaluating claims of the biological significance of lowly expressed miRNAs.

## Results

### Constructing sensitive reporters of endogenous miRNA activity

**Figure 1:**
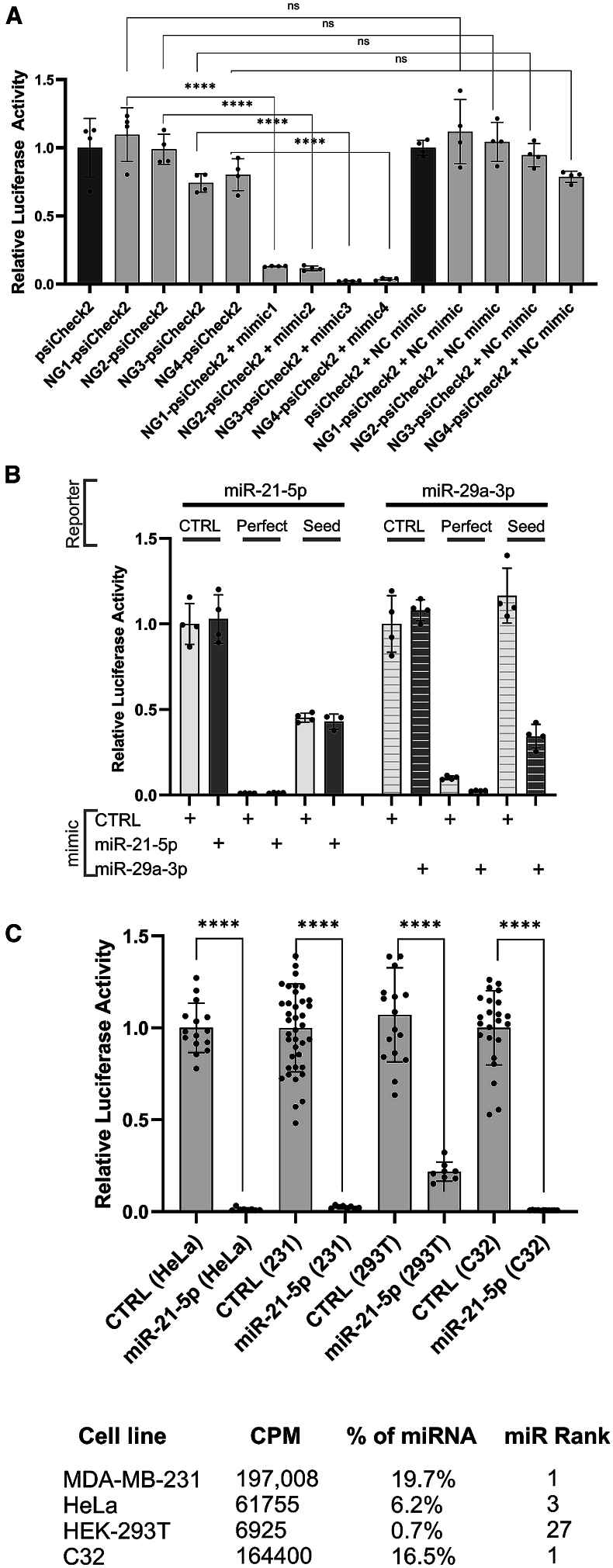
Constructing sensitive reporters of endogenous miRNA activity (A) psiCheck2 vectors were modified to include 21 nt sequences not present within the human genome (“NG1-4”) downstream of renilla. Cells were then co-transfected with these vectors and small RNAs of complementary sequence (“mimic 1–4”) or a negative control “NC” mimic. Dual Renilla luciferase reporter assays were then conducted 48 h post transfection. (B) Renilla luciferase reporters were constructed in which the reporter was either unmodified (CTRL), or where sites for miR-21-5p or miR-29a-3p were introduced into the Renilla luciferase 3′UTR that are either fully complementary across the entire length of the 21 nt binding interface (“perfect”) or complementary only across an 8 nt seed-site (“seed,” nt 2–9 inclusive relative to the 5′ end of the miRNA). Dual Renilla luciferase reporter assays were conducted, co-transfecting the Renilla luciferase expression plasmid along with negative control miRNA mimic, or mimics to miR-21 and miR-29a. (C) The miR-21-5p reporter was transfected into 4 separate cell lines and reporter activity determined relative to luciferase. miR-21-5p expression in each cell line, as determined by small-RNA-seq is shown. Significance is calculated by unpaired t-test; ∗p=<0.05; ∗∗p=<0.01; ∗∗∗p=<0.001; ∗∗∗∗p=<0.0001. “NS” = not significant.

Despite nearly 200,000 miRNA publications, the number of genuine miRNAs remains controversial. Many annotated miRNAs may be artifacts – either degradation products or imprecisely processed RNAs that do not generate functional regulatory modules.^7^^,^^8^^,^^21^^,^^23^^,^^24^^,^^25^ In virtually every study, function is demonstrated (at least in part) by the transfection of a miRNA mimic and the resultant repression of a reporter gene that possesses a region of sequence complementarity to the miRNA. However, such an assay does not indicate anything other than the effects of over-expression, as artificial “miRNAs” that are not encoded within the genome, are nevertheless able to strongly downregulate reporters with matching complementary sequences (Figure 1A).

As exogenous expression is therefore of no benefit in establishing functionality, we sought to establish the minimal level of expression that is required to detect endogenous miRNA activity, then interrogate large public sequencing repositories to assess how often individual miRNAs meet these thresholds. To do this, we first sought to establish highly sensitive reporters, rationalizing that although mammalian miRNAs rarely interact with their targets with full sequence complementarity, such reporters should be highly sensitive as the extensive miRNA-target interaction interface leads to stronger binding and the unmasking of AGO2’s endogenous slicer activity (directly cleaving the target mRNA).^26^ To confirm this strategy, both full complementary (highly sensitive) and seed-only (endogenous-like) binding sites were engineered downstream of a Renilla luciferase reporter for two highly expressed miRNAs; miR-21-5p (the 3^rd^ highest expressed miRNA in HeLa cells - 62k cpm) and miR-29-3p (the 12^th^ highest expressed miRNA - 25k cpm). In both cases, the activity of the perfect reporter was vastly decreased relative to both unmodified Renilla luciferase and the seed-only reporter. In the case of miR-21-5p, exogenous miR-21-5p has no additional effect, presumably as cellular AGO was already sufficiently loaded with miR-21 to maximally suppress activity of the introduced reporter. In the case of miR-29a-3p, additional miRNA was able to exert further repression (Figure 1B). Extreme sensitivity of the full-complementarity reporters was further demonstrated upon miR-21-5p reporter transfection across multiple cell lines. In three cell lines (where miR-21-5p is between the 1^st^ and 3^rd^ most abundant miRNA), reporter activity is completely eliminated, whilst in 293T cells (where miR-21-5p is the 27^th^ most abundant miRNA), reporter activity was diminished by approximately 80% (Figure 1C). These results confirm fully complementary reporters are far more sensitive than seed-only reporters (as one would expect) and that luciferase/reporter activity is strongly depleted by endogenous miRNAs, thus providing a highly sensitive artificial system by which to gauge endogenous miRNA-mediated repression.

As the degree to which a target can be repressed is a balance between the level of target and repressor, we further sought to establish optimal levels of reporter transfection such that the amount of reporter yielded reproducible results, but where the amount of reporter was minimised to ensure maximal sensitivity (Figure S1). To do this, titrated amounts of 4 fully complementary miRNA reporters were transfected against miRNAs which are not expressed in HeLa cells (miR-382), moderately expressed (miR-126, the 71^st^ most abundant miRNA in HeLa cells) and highly expressed (miR-27a and miR-21, the 9^th^ and 3^rd^ most abundant miRNAs respectively). On the basis of these data, 5 ng psiCHECK2 was selected for future experiments. Additional fully complementary reporters were then constructed to measure the activity of miRNAs, ranging from those with little to no endogenous expression, through to those that are moderately and highly expressed.

### Endogenous miRNA activity is not detected for miRNAs expressed below several hundred counts per million

**Figure 2:**
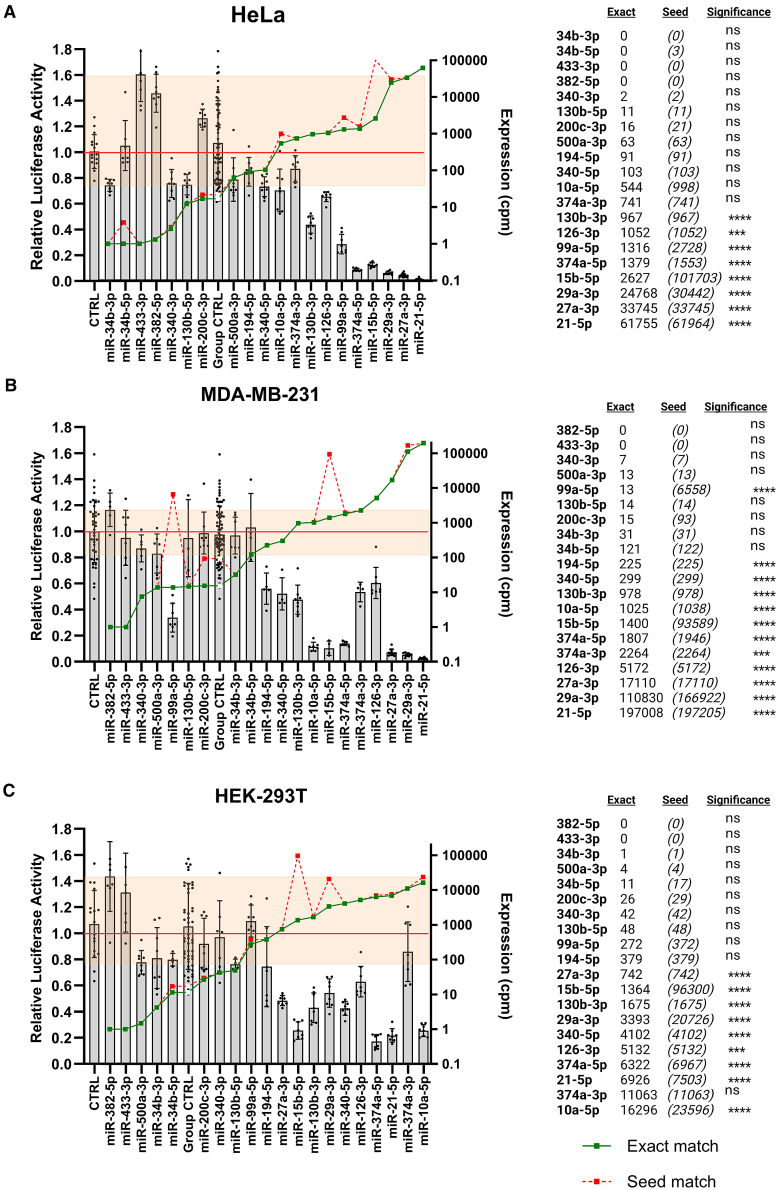
Endogenous activity is not detected for miRNAs expressed below 200 cpm Dual luciferase reporter assays were conducted in (A) HeLa, (B) MDA-MB-231, and (C) HEK-293T cells in which Renilla luciferase reporters that were perfectly complementary to the miRNAs indicated had been transfected. Relative luciferase activity is shown on the left axis, expression level (cpm) for the miRNA in question is shown on the right (green line). The dotted red line indicates the sum expression of all miRNAs whose seed site would be predicted to also target the reporter (as indicated by TargetScan, requiring the matching of nt 2–7, relative to the 5′ end of the miRNA). The shaded yellow section indicates the upper and lower bounds of relative luciferase activity for reporters for miRNAs expressed at <20 cpm. Significance is calculated by unpaired t-test against the Group control; ∗p=<0.05; ∗∗p=<0.01; ∗∗∗p=<0.001; ∗∗∗∗p=<0.0001. “NS” = not significant. The “CTRL” reporter is the empty dual luciferase vector. “Group CTRL” is the summation of all measurements for reporters of miRNAs expressed at <20 cpm and is located in each graph immediately after the last of these reporters.

Using a dual-luciferase reporter assay, the activity of 20 reporter genes engineered to be responsive to the presence of the miRNAs indicated were quantitatively assessed in three cell lines: HeLa (Figure 2A), MDA-MB-231 (Figure 2B), and HEK-293T (Figure 2C). There is a clear trend that miRNA reporter activity decreases as the endogenous expression of the corresponding miRNA increases, indicating reporters provide a readout of miRNA presence. However, reporter systems display intrinsic variability, on account of such factors as the presence of cryptic sites for miRNAs other than that to which they were designed, the sequestering effects of the endogenous target pool or the creation of binding sites for other RNA-binding proteins of which there are many that associate with 3′UTRs.^27^ Accordingly, we see variation in reporter activity, even in some cases where a reporter has been designed to a miRNA which is not expressed and for which there are no other obvious miRNA binding candidates. For this reason, the “group CTRL” bar has been added, which is the summation of measurements for all reporters designed against miRNAs for which detectable endogenous activity is expected to be negligible (<20 cpm). The shaded horizontal yellow bar indicates the upper and lower bounds of relative luciferase activity for reporters for miRNAs expressed at <20 cpm. Statistical significance was then calculated between each other reporter and this group control. This is indicated in the adjacent table (“Significance”), as are read counts of the miRNA to which the reporter was designed (“Exact”) and read counts of other miRNA family members that share the same seed site (“Seed”) and thus, would be expected to target the reporter, but to do so less efficiently (in a non-siRNA like manner). This is why for example, the miR-99a-5p reporter is strongly suppressed in MDA-MB-231 cells, as although miR-99a-5p expression is very low, its seed-matched counterpart miR-99b-5p, is present at far higher levels.

In all cases where a miRNA is expressed above 1000 cpm, statistically significant repression was detected, with the exception of miR-374a-3p in HEK293T cells. We note that both miR-374a-3p and miR-126-3p are consistently less active than other miRNAs expressed at similar levels (Figures 2A–2C). This shows that the high expression of a miRNA is not the only determinant of function and that in some cases, other factors, such as the number of competing target sites in other transcripts^28^^,^^29^ or AGO-binding affinity, may be important. Consistent with this, in data derived from two separate cell lines (mammary epithelial HMLE cells and their mesenchymal “mesHMLE” derivative created by prolonged exposure to TGF-β^30^), the relative efficiency with which miR-374a-3p (and to a lesser extent miR-126-3p) is co-precipitated with AGO relative to its expression level, was lower than the other miRNAs that exerted suppressive effects on their respective reporters (Figure S2).

In no instance was statistically significant repression detected for any reporter designed against a miRNA expressed at less than 200 cpm. The capacity to detect the repressive activities of miRNAs expressed between 200 and 1000 cpm was variable. These findings are in strong agreement with a previous study, where 80% of miRNAs expressed at >1000 cpm showed suppressive activity in a fluorescent-based reporter screen, whilst repression was only detected for <2% of miRNAs expressed at <100 cpm, which the authors speculated may have been false-positives as no repressive activities were detected for any miRNA expressed below this range in a second cell line.^18^

### Many miRNAs are never expressed at a sufficient level for endogenous functionality

**Figure 3:**
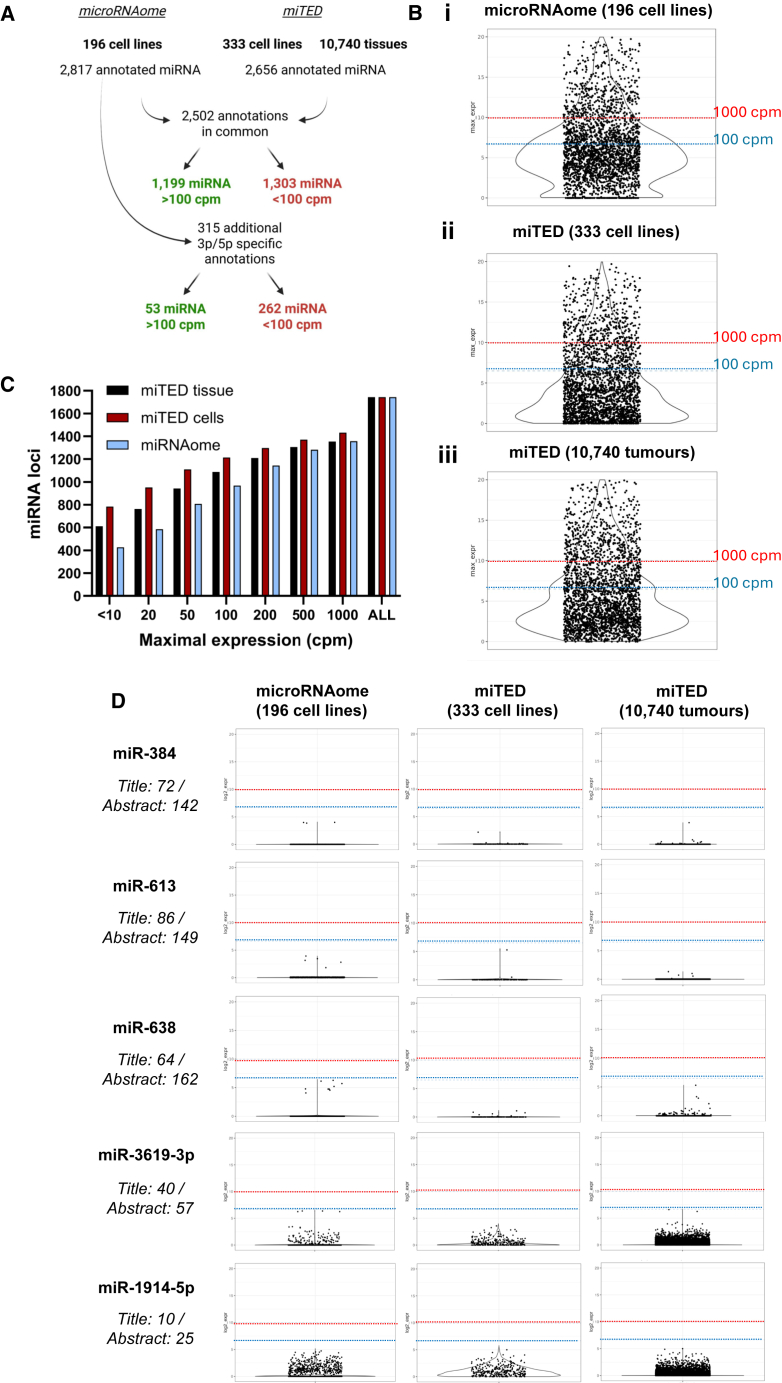
Assessment of maximal miRNA expression across cell lines and tissues (A) The maximal cpm expression for each annotated miRNA is shown across all small RNA-seq data present in the microRNAome^20^ and miTED^19^ resources. (B) (i) The microRNAome contains 2406 sequencing runs of diverse tissue representing 196 distinct cell types whilst miTED contains data derived from B (ii) 333 cell lines and B (iii) 10,740 tissues drawn from 176 sites. Log2 values are graphed with dotted lines indicating 100 (blue) and 1000 (red) cpm. (C) Cumulative numbers of miRNA loci (from a total of 1,744 loci) and their maximal expression in any cell or tissue from amongst the 3 aggregated datasets analyzed. (D) Expression for individual lowly expressed miRNAs across cell lines and tissues. Title/Abstract refers to the number of publications featuring that miRNA name in the article title or abstract by PubMed search. miR-3619 and miR-1914 are included as representative examples of lowly expressed miRNAs listed within MirGeneDB, a manually curated, high confidence listing of miRNAs.

Our data, and others,^17^^,^^18^ show that miRNAs expressed below 200 cpm exert no discernible repression of reporter genes, even when the reporters are specifically designed to be more sensitive than endogenous targets. Consequently, we examined public small-RNA sequencing data to see how often miRNAs fail to meet a minimum threshold of expression where target suppression can be expected. To do so, we set a deliberately conservative threshold requiring any given miRNA to be expressed at a minimum of 100 cpm in at least one of the thousands of samples examined (Figure 3A). Figure 3 presents the results of such an analysis, with each dot representing the maximal expression of each miRNA across all the samples contained within the “microRNAome” and “miTED” datasets. “MicroRNAome” represents 2,817 annotated miRNAs and their maximal expression among 196 distinct tissue types measured across a total of 2,406 sequencing runs (Figure 3Bi).^20^ “miTED” contains 2,656 miRNAs, a slightly lower number than microRNAome, as distinct 3p and 5p designations were assigned to fewer miRNAs. The maximal level of expression for each miRNA across 333 cell lines and 10,740 tissue samples (Figures 3Bii,iii), representing 176 different tissues from both normal and pathologic states, is shown. Importantly, 1,565 miRNAs are never expressed above 100 cpm in any cell or tissue. Represented another way, this means 876 miRNA-encoding loci (out of 1,744 human loci annotated in miRBase) do not produce any miRNAs above this threshold level of expression, including 353 loci from which small RNAs are never detected at more than 10 cpm (Figure 3C).

In addition to ongoing controversies regarding the false annotation of degradome products and small RNAs which are imprecisely processed or inefficiently bound to AGO,^7^^,^^8^^,^^21^^,^^23^ the simple fact that many miRNAs are rarely (if ever) expressed at a level where their function is detectable with highly sensitive reporters calls into the question the significance of much of the miRNAome. Even so, there are >2,600 publications where lowly expressed miRNAs (<100 cpm) are named in the title, and >6,500 publications where such miRNAs feature in the abstract. These are listed in Table S4. Representative expression profiles are shown for several highly published, lowly expressed miRNAs (Figure 3D). miRs -3619-3p and -1914-5p are included as they are not expressed above 100 cpm in any cell or tissue sample (nor are miRNAs derived from their respective opposing hairpin arms), though they are present in MirGeneDB; a manually curated database that applies stringent criteria based upon the precision of processing and other features expected of bona fide miRNAs.^21^ The existence of 24 lowly expressed miRNAs among the 505 distinct miRNAs listed in MirGeneDB suggests a discrepancy between the designation of bona fide miRNAs that are the product of the canonical miRNA biogenesis pathway and those that reach a sufficient level of expression where their function is likely important.

### Lowly expressed microRNAs derive from a more recent evolutionary origin

The evolutionary origins of miRNAs can be traced back to early metazoans, with gene duplications and divergence giving rise to the more than 1,700 human miRNA loci currently annotated. The observation that a substantial proportion of miRNAs are expressed at levels too low to exert detectable regulatory effects is surprising if these lowly expressed miRNAs share a deep evolutionary history and have been conserved over hundreds of millions of years. Conversely, if these poorly expressed miRNAs represent more recent evolutionary innovations whose functional roles are not yet established, or if they are simply mis-annotations, then their apparent lack of biological relevance is less unexpected.

**Figure 4:**
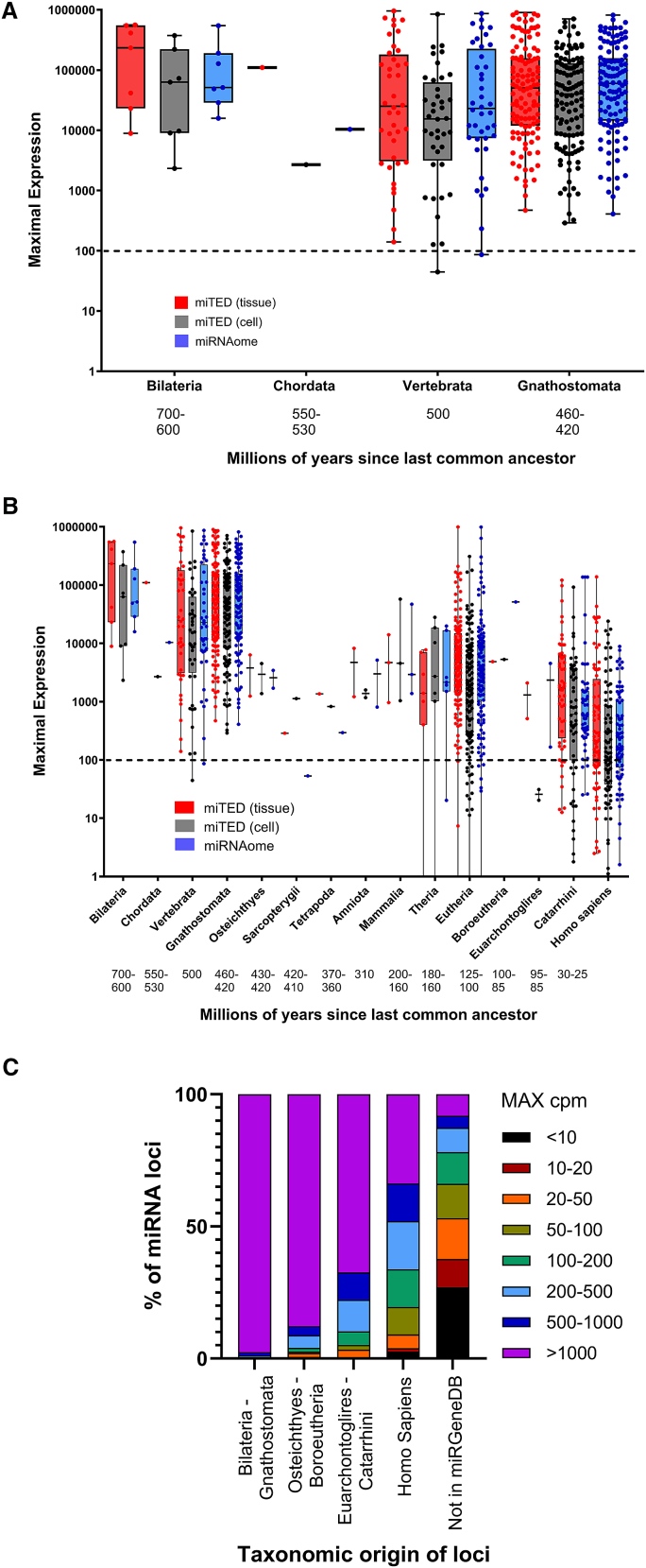
Evolutionarily conserved microRNAs are more highly expressed (A and B) Maximal expression of small RNAs derived from each miRNA locus (dots) grouped by the taxonomic origin of each locus. Time since divergence from a common ancestor is indicated. (C) Percentage of miRNA loci and their maximal expression is shown, grouped again by taxonomic origin.

To investigate these possibilities, we grouped miRNAs cataloged in miRGeneDB^7^^,^^21^ - which are presumed functional because they meet stringent processing criteria - according to their inferred taxonomic origin. We then determined each locus’s maximal miRNA expression across all cell and tissue samples in the miTED and microRNAome resources (∼13,000 samples). For miRNAs with ancient origins (>420 million years ago), all exceed a conservative threshold of 100 cpm in at least one sample, with most expressed well above this level (Figure 4A). The two dots in Figure 4A below the 100 cpm threshold represent miRNAs whose expression is above the threshold in other datasets. In contrast, miRNAs with more recent evolutionary origins tend to show progressively lower maximal expression (Figure 4B). Notably, among miRNAs specific to *Homo sapiens*, 20% fail to reach the 100 cpm threshold in any dataset.

In comparison, miRNAs annotated in miRBase but absent from miRGeneDB - likely representing RNA degradome fragments or imprecisely processed transcripts that have not been refined by evolution into genuine miRNAs^7^^,^^21^^,^^31^ - are far more likely to be undetectable at functionally meaningful levels. 66% of these non-miRGeneDB “miRNAs” never reach 100 cpm in any sample, and 27% do not exceed 10 cpm (Figure 4C; Tables S1, S2, and S3).

Taken together, these data strongly support the contention that miRNAs failing to meet a biologically significant level of expression are largely made up of falsely annotated “miRNAs,” and to a lesser extent, miRNAs that have recently emerged but may yet to evolve important functions.

## Discussion

In exactly the same way one can introduce synthetic siRNAs that do not exist in nature to silence genes, one can introduce sequences representing miRNAs that will repress targets, even if the miRNA itself is not present in the endogenous context (Figure 1A). Therefore, publications that are reliant upon miRNA over-expression can describe apparent phenotypic effects and demonstrate gene targeting, without ever establishing an endogenous role.

Based on our findings in the highly sensitive reporter system we have created, miRNAs that are present at less than 200 cpm (0.02% of total miRNA expression, listed in Table S5A) do not show any evidence of detectable function. This is in agreement with a previous report in which the repression of fluorescent reporters to 291 miRNAs was detected for 80% of miRNAs expressed at >1000 cpm, but only for 2% of miRNAs expressed at <200 cpm (of which no repressive effects were then corroborated in a second cell line).^18^ It is not that such miRNAs are necessarily inherently incapable of function, but rather the proportion to which they represent the gene targeting pool is so small their silencing, even of highly sensitive reporters, are simply not discernible. The surprise, however, is that for over half of all annotated miRNAs, their endogenous expression never reaches this level, at least across the extensive datasets examined. This, in turn, calls into question the significance of thousands of publications of which they are the collective subject. While not the purview of this report, it should be noted there are also ongoing debates about differentiating genuine miRNAs from misannotated products of the RNA degradome,^6^^,^^7^^,^^8^^,^^32^^,^^33^^,^^34^^,^^35^ and it is likely that in addition to simple expression levels, misannotated RNA fragments may also account for a number of “miRNAs” which are non-functional, regardless of their expression level. Consistent with this, a large proportion of “miRNAs” annotated in miRBase but not miRGeneDB (and thus are more likely to be degradome products or imprecisely processed), fail to attain levels of expression which we believe are of biological significance (Figure 4C).

We note there are limitations of this study. One is that it remains possible there is some cell context in which a given miRNA is expressed at a sufficient level and where it plays an important role, but is not captured in the data analyzed. This could include individual cells, though technological limitations so far preclude the measurement of miRNA expression by single-cell RNA sequencing. The miRNA lsy-6 for example controls neuronal left/right asymmetry of chemosensory receptor expression through its strong expression in less than ten neurons and it is entirely possible that among the lowly expressed miRNAs we report, similar examples of highly specific expression may yet be uncovered.^36^ Similar arguments are proposed regarding lncRNAs, where thousands of lncRNAs are detected at low levels in bulk-sequencing, but where examples of highly cell-specific expression have been noted.^37^ It is impossible to fully eliminate this possibility, but is worth noting that the small RNA sequencing we analyze is extensive (>13,000 tissue samples and cell lines).^19^^,^^20^ Another limitation is that one cannot definitively establish a point at which a miRNA is or is not capable of gene suppression. This is because there are methodological limitations in the detection of miRNA function, though the fully complementary reporter system is designed to be more sensitive than one would expect when compared with most endogenous targets. Further, our “non-functional” cutoff of 100 cpm is deliberately conservative as both our data (Figure 2), and that derived from an independent reporter system,^18^ suggests that a threshold of ∼200 cpm may be a level of expression closer to that where reporter repression becomes discernible. If anything, we therefore expect we are over-estimating miRNA function rather than under-estimating it.

Another potential short-coming is that miRNAs can work co-operatively and thus, by focusing on the actions of miRNAs individually, one can miss the contribution they make collectively.^38^ Such collective actions may be through co-operative binding between miRNAs at nearby sites,^39^^,^^40^ via multiple miRNAs independently targeting the same transcript, or multiple miRNAs targeting different transcripts within common pathways to exert pathway-level effects.^41^^,^^42^^,^^43^^,^^44^^,^^45^^,^^46^^,^^47^ Further, the contribution of miRNAs may be to serve a buffering role in gene expression whose individual effects may be difficult to discern across a broad range of modestly suppressed genes.^48^^,^^49^^,^^50^^,^^51^ It is extremely difficult to exclude the possibility of such actions, and so we cannot definitively claim that any given miRNA is simply non-functional. We do note, however, that among the thousands of publications in which lowly expressed miRNAs are a focus, almost never are claims made for their subtle, buffering, and/or collective roles. This means that even if lowly expressed miRNAs do in fact play subtle and collective roles that are difficult to discern, it still remains true that their biological functions have been vastly over-stated. It is also worth noting that the vast majority of reports involving lowly expressed miRNAs are not undertaken in obscure cells or tissues unrepresented in the datasets analyzed here, but rather take place in common cell lines where we know the endogenous levels of these miRNAs to be very low. As such, the conclusions of this study should not be taken as a binding repudiation of the function of any specific miRNA in all circumstances, but do shift the burden of proof to the researcher working with lowly expressed miRNAs to demonstrate endogenous function. By definition, claims of endogenous function cannot include observations made under conditions of exogenous miRNA expression (Figure 1A).

Considerations of miRNA expression are not “niche” as slightly more than half of annotated miRNA-encoding loci fail to produce any small RNAs that meet the 100 cpm threshold in any of the >13,000 cells/tissues examined. We note a tendency for lowly expressed miRNAs to be poor substrates for DROSHA processing^22^ (Figure S3; Table S5B), and for more recently emerged miRNA loci to produce more lowly expressed miRNAs (Figures 4B and 4C). Amongst miRNAs whose processing meets the stringent criteria for miRGeneDB listing, all whose taxonomic origins can be traced back hundreds of millions of years are expressed above 100 cpm, though 20% of human-specific miRNAs are not. In such instances, these miRNAs may be yet to evolve important functions and may be described as “transitional”; a term coined to describe small RNAs in the process of evolving miRNA-like activity, but whose gene silencing roles are currently limited.^52^ Importantly, 66% of “miRNAs” excluded from miRGeneDB do not meet minimal expression, while 27% were not detected above 10 cpm. As such, by simply examining the expression of annotated miRNAs, our data support arguments that call into question the annotation and/or function of over half of the annotated human miRNAome. A complete list of lowly expressed miRNAs, along with the evolutionary origin of miRNA-encoding loci and the efficiency by which DROSHA processes their respective hairpins (obtained from^22^), is provided as a resource to researchers and reviewers in Table S5B. Complete data for miRNA expression across the miRNAome and miTED repositories are provided in Tables S1, S2, and S3. We urge particular caution be brought to any study seeking to assess their function.

### Limitations of the study

This study infers the expression threshold required for miRNAs to elicit detectable target suppression using highly sensitive artificial reporters. As these reporters are more responsive than typical endogenous targets, the required miRNA levels may, if anything, be overestimated. All analyses were conducted with single miRNAs and single reporters, potentially overlooking combinatorial effects where even lowly expressed miRNAs might contribute to biological outcomes as part of a broader network. That said, most published claims about miRNA function present them as standalone regulators, not subtle contributors within complex interactions. Additionally, while expression was assessed across thousands of cell lines and tissues, rare, transient, or developmentally restricted cell populations may be underrepresented. It remains possible that some miRNAs deemed lowly expressed may reach functional levels in such contexts. However, most functional claims in the literature do not invoke such specialised cellular states.

## Resource availability

### Lead contact

Requests for further information and resources should be directed to and will be fulfilled by the corresponding author, Cameron Bracken (cameron.bracken@unisa.edu.au).

### Materials availability

All reporter constructs generated in this study are available from the lead contact without restriction.

### Data and code availability

• Small RNA-seq data (from Figure 2) can be downloaded from the GEO repository: GSE303781.
• This article does not report original code.
• Any additional information required to reanalyze the data reported in this article is available from the lead contact upon request.

## Acknowledgments

This work was supported by funding from the 10.13039/501100000923Australian Research Council to C.P.B (FT190100544, DP190103333), the 10.13039/501100000925National Health and Medical Research Council of Australia (NHMRC) to P.A.G. (1128479, 1164669), the Cancer Council Beat Cancer Project to C.P.B (PRF2518), the 10.13039/501100001026National Breast Cancer Foundation to P.A.G. (IIRS18147 and IIRS0098). the 10.13039/100009727Hospital Research Foundation to P.A.G. (2023-S-DTFA-001-83100), and the Worldwide Cancer Research Foundation (WCR-19-0300). K.A.P. was supported by the Col Reynolds Fellowship from 10.13039/501100018675The Kids' Cancer Project. P.A.G. was supported by a Principal Cancer Research Fellowship awarded by Cancer Council’s Beat Cancer project on behalf of its donors, the state Government through the Department of Health, and the Australian Government through the Medical Research Future Fund. The authors would like to thank Prof Greg Goodall for his critical reading of the article and suggestions.

## Author contributions

S.A.K performed the cloning and luciferase reporter assays. J.M.B led the bioinformatic work supported by K.A.P. P.A.G provided oversight, intellectual contribution and critical editing. C.P.B directed the project, led data analysis and wrote the article.

## Declaration of interests

The authors declare no competing interests.

## STAR★Methods

### Key resources table

**Table:** 

REAGENT or RESOURCE	SOURCE	IDENTIFIER
**Chemicals, peptides, and recombinant proteins**		

Trizol Reagent	Ambion	Cat# 15596018
Lipofectamine™ 2000 Transfection Reagent	Invitrogen	Cat# 11668019
T4 DNA Ligase	New England BioLabs (NEB)	Cat# M0202S
NotI-HF restriction enzyme	New England BioLabs (NEB)	Cat# R3189S
XhoI restriction enzyme	New England BioLabs (NEB)	Cat# R0146S

**Critical commercial assays**		

Dual-Luciferase® Reporter Assay System	Promega	Cat# E1980
Bioanalyzer Small RNA Kit	Agilent	Cat# 5067-1548
QIAseq miRNA Library Kit	Qiagen	Cat# 331502
Qubit DNA HS Assay Kit	Invitrogen	Cat# Q32852

**Deposited data**		

GEO repository	–	GSE303781

**Experimental models: Cell lines**		

MDA-MB-231	ATCC	HTB-26
HEK293T	ATCC	CRL-3216
HeLa	ATCC	CCL-2

**Oligonucleotides**		

Custom oligonucleotides for reporter cloning. See Table S6.	Integrated DNA Technologies (IDT)	N/A
Oligos for reporter constructs. See Table S6.	Integrated DNA Technologies (IDT)	N/A
miRNA mimics. See Table S6.	Gene Pharma	N/A

**Recombinant DNA**		

psiCHECK-2 Dual Luciferase Vector	Promega	Catt# C8021

### Experimental model and study participant details

HEK-293T, HeLa and MDA-MB-231 cells were obtained from American Type Culture Collection (ATCC) and cultured in DMEM (Dulbecco’s Modified Eagle Medium-Gibco) supplemented with 10% fetal calf serum (FCS; Cytiva, #SH30071.03). All cell lines were incubated at 37°C in a humidified atmosphere containing 5% CO_2_, with mycoplasma testing performed regularly.

### Method details

#### Reporters construct design and cloning

The PsiCHECK2 dual luciferase reporters (Promega, #C8021) were designed to incorporate a single copy of a target site that perfectly complements small RNA sequences from miRBase.^5^ Oligonucleotides (IDT technologies) were created with overhangs for XhoI (New England BioLabs,#R0146S) and NotI-HF (New England BioLabs, #R3189S), which were annealed and ligated using T4 DNA Ligase (NewEngland BioLabs, #M0202S) into psiCHECK2 vector using the same restriction sites. Primers used to construct the reporters and miRNA mimics are listed in Table S6.

#### Luciferase reporter assays

HeLa and HEK-293T cells were plated in 96-well plates at densities of 10,000 and 20,000 cells per well, respectively, while MDA-MB-231 cells were seeded in 24-well plates at a density of 100,000 cells per well 1 day before transfection. Prior to transfection, the medium was changed, and the cells were co-transfected with 5 ng of miRNA psiCHECK2 reporter constructs using Lipofectamine 2000 Transfection Reagent (Invitrogen, #11668019), following the manufacturer’s instructions. The medium was again changed 6 h after transfection. After 48 h, luciferase activity assay was conducted using the Dual-Luciferase Reporter Assay System (Promega, #E1980) on a GloMax-Multi Detection System (Promega), in accordance with the manufacturer’s guidelines. The relative luciferase activity was determined by calculating the ratio of Renilla to Firefly luciferase, with the Firefly luciferase gene expressed from the same vector serving as an internal control, while cells transfected with the empty psiCHECK2 vector acted as a control group.

#### RNA extraction and small RNAseq

For each cell line RNA was extracted according to Trizol Reagent (Ambion, #15596018) manufacturer instructions. The quantity and of the RNA samples were checked using nanodrop Qubit HS RNA (Invitrogen, #Q32852) and Bioanalyser small RNA assay (Agilent, #5067-1548). Libraries were generated using 1 μg of the total RNA via QIAseq miRNA kit (Qiagen, # 331502) with 14 cycles of amplification. Amplified barcoded libraries were then size selected using auto gel-purification Pippin prep 3% agarose (SAGE science) which targets ranges from 100 to 250bp. Libraries (size between 180 and 190bp) were then confirmed by Qubit HS DNA and Bioanalyzer HS DNA assay for size and concentration. The libraries were then pooled together in equimolar amounts and sequenced using an Illumina MiSeq Reagent Kit v3 kit in accordance with the manufacturer’s guidelines. Methodological details for the AGO:RNA co-immunoprecipitation (Figure S2) and the growth of HMLE and mesHMLE cell lines are fully detailed in Orang et al.^23^

#### Data sources for bioinformatic assessment

Small RNA sequencing data was obtained from miTED^19^ and the microRNAome^20^ and deposited in Tables S1, S2, and S3. The designation of hairpins as good or poor DROSHA substrates was taken from Kim et al.^22^

### Quantification and statistical analysis

All luciferase assays were conducted in four technical replicates per condition and repeated in at least three independent biological replicates. Data are presented as mean ± standard error of the mean (SEM). Statistical significance was determined using an unpaired, two-tailed Student’s t test. Graphs were generated using GraphPad Prism version 9.0. A *p*-value <0.05 was considered statistically significant.

## Supplemental information

Document S1. Figures S1–S3 and Table S6

Table S1. miRNA expression in cell lines contained within the miRNAome dataset

Table S2. miRNA expression in cell lines contained within the miTED dataset

Table S3. miRNA expression in tissue samples contained within the miTED dataset

Table S4. Publication counts for lowly expressed miRNAs

Table S5. S5A: Maximal miRNA expression levels across datasets listed in Tables S1-S3 S5B: DROSHA processing efficiency taken from Kim et al., Mol Cell 2021; S5C: Evolutionary conservation of miRNA families.
