# Supplementary material for: Half of annotated human microRNAs are expressed at levels of questionable biological significance
Source: iScience. 2025 Aug 25;28(9):113445. doi: 10.1016/j.isci.2025.113445 (PMC12450571; doi:10.1016/j.isci.2025.113445)
Supplement: Document S1. Figures S1–S3 and Table S6 [file mmc1.pdf]

iScience, Volume *28*

Supplemental information

Half of annotated human microRNAs are expressed at levels of questionable biological significance

Saba Ataei Kachooei, Julie M. Bracken, Katherine A. Pillman, Philip A. Gregory, and Cameron P. Bracken

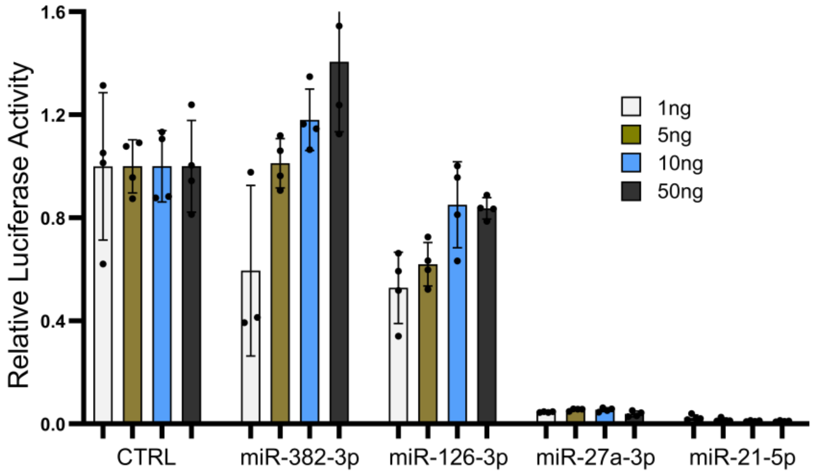

Cell line	CPM	miR Rank
miR-382	0	-
miR-126	1052	71
miR-27a	42,200	9
miR-21	61,800	3

Supplementary Figure 1 – Optimising sensitive reporters
Renilla-luciferase vectors constructed to report on the activities of the miRNAs indicated were transfected into HeLa cells at 1, 5, 10 and 50ng. Endogenous miRNA expression in HeLa cells is indicated.

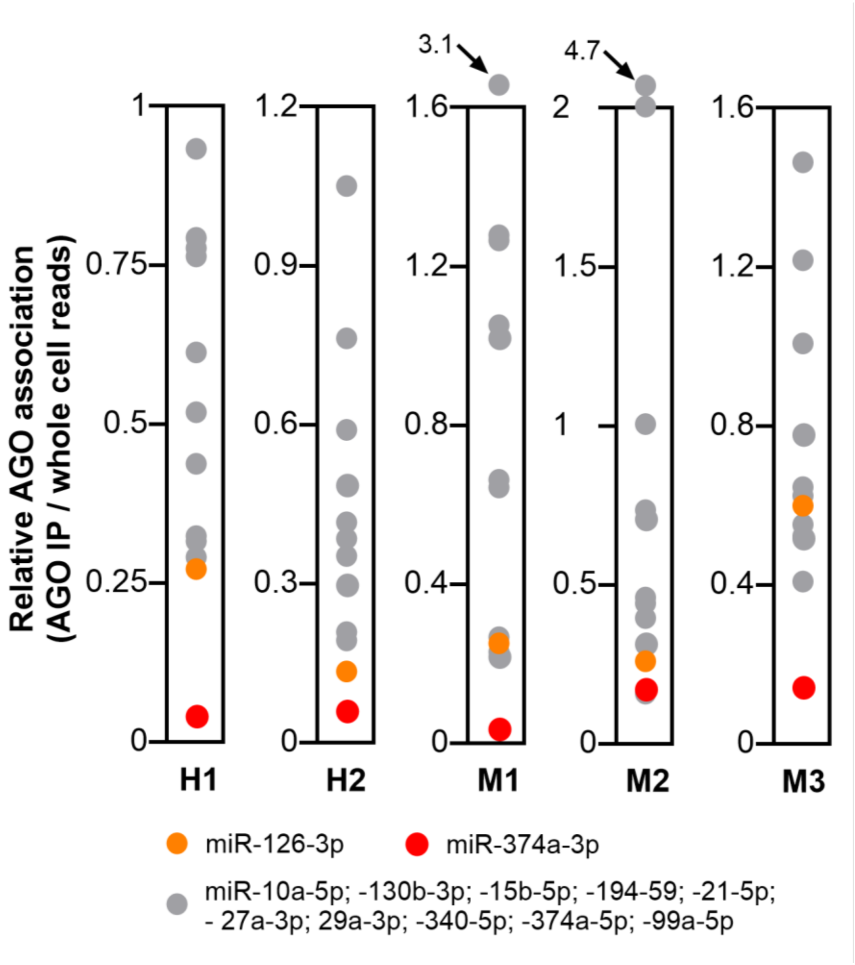

Supplementary Figure 2. miR-374a-3p and miR-126-3p are consistently less efficiently co-precipitated with AGO than are typical functional miRNAs

In HMLE cells (H), and their mesenchymal mesHMLE derivative (M), the relative AGO-binding efficiency of different miRNAs is estimated by calculating the ratio of miRNA sequenced after AO co-immunoprecipitation versus the amount of miRNA detected in whole cell small RNA sequencing. The lower the ratio, the less miRNA is associated with AGO. Grey dots represent each miRNA from Figure 2 for which repressive activity was detected. Orange dots indicate miR-126-3p and red dots miR-374a-3p (which are less repressive than one might expect given their level of expression, Figure 2).

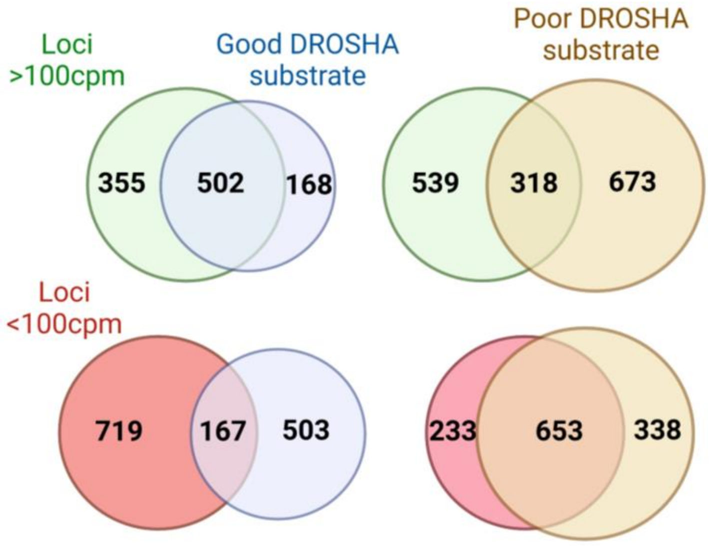

Supplementary Figure 3. The hairpins of lowly expressed microRNAs tend to be poor substrates for DROSHA processing.

Hairpins designated as good or poor substrates for processing in an in vitro cleavage assay (Kim et al, Mol Cell 2021) were cross-referenced with lowly (<100 cpm) or highly (>100 cpm) miRNAs across all datasets.

Micro-RNA reporters		Sequence 5'→ 3'
miR-21-5p (Perfect)	F	TCGAGATTTAAATTCAACATCAGTCTGATAAGCTAGC
	R	GGCCGCTAGCTTATCAGACTGATGTTGAATTTAAATC
miR-21-5p (Partial)	F	TCGAGATTTAAATCTCGACTCAGCTCAATAAGCTCGC
	R	GGCCGCGAGCTTATTGAGCTGAGTCGAGATTTAAATC
miR-29a-3p (Perfect)	F	TCGAGATTTAAATTAACCGATTTCAGATGGTGCTAGC
	R	GGCCGCTAGCACCATCTGAAATCGGTTAATTTAAATC
miR-29a-3p (Partial)	F	TCGAGATTTAAATCATTAGAATTTGCTCTGGTGCTCGC
	R	GGCCGCGAGCACCAGAGCAAATTCTAATGATTTAAATC
miR34b-3p	F	TCGAGATTTAAATATGGCAAGTGGAGTTAGTGATTGGC
	R	GGCCGCCAATCACTAACTCCACTTGCCATATTTAAATC
miR34b-5p	F	TCGAGATTTAAATCAATCAGCTAATGACACTGCCTAGC
	R	GGCCGCTAGGCAGTGTCATTAGCTGATTGATTTAAATC
miR433-3p	F	TCGAGATTTAAATACACCGAGGAGCCCATCATGTAGC
	R	GGCCGCTACATGATGGGCTCCTCGGTGTATTTAAATC
miR382-5p	F	TCGAGATTTAAATCGAATCCACCACGAACAACTTCGC
	R	GGCCGCGAAGTTGTTCGTGGTGGATTCGATTTAAATC
miR340-3p	F	TCGAGATTTAAATGCTATAAAGTAACTGAGACGGAGC
	R	GGCCGCTCCGTCTCAGTTACTTTATAGCATTTAAATC
miR130b-5p	F	TCGAGATTTAAATGTAGTGCAACAGGGAAAGAGTGC
	R	GGCCGCACTCTTTCCCTGTTGCACTACATTTAAATC
miR200c-3p	F	TCGAGATTTAAATTCCATCATTACCCGGCAGTATTAGC
	R	GGCCGCTAATACTGCCGGGTAATGATGGAATTTAAATC
miR500a-3p	F	TCGAGATTTAAATCAGAATCCTTGCCCAGGTGCATGC
	R	GGCCGCATGCACCTGGGCAAGGATTCTGATTTAAATC
miR194-5p	F	TCGAGATTTAAATTCCACATGGAGTTGCTGTTACAGC
	R	GGCCGCTGTAACAGCAACTCCATGTGGAATTTAAATC
miR340-5p	F	TCGAGATTTAAATAATCAGTCTCATTGCTTTATAAGC
	R	GGCCGCTTATAAAGCAATGAGACTGATTATTTAAATC
miR10a-5p	F	TCGAGATTTAAATCACAAATTCGGATCTACAGGGTAGC
	R	GGCCGCTACCCTGTAGATCCGAATTTGTGATTTAAATC
miR374a-3p	F	TCGAGATTTAAATAATTACAATACAATCTGATAAGCGC
	R	GGCCGCGCTTATCAGATTGTATTGTAATTATTTAAATC
miR130b-3p	F	TCGAGATTTAAATATGCCCTTTCATCATTGCACTGGC
	R	GGCCGCCAGTGCAATGATGAAAGGGCATATTTAAATC
miR126-3p	F	TCGAGATTTAAATCGCATTATTACTCACGGTACGAGC
	R	GGCCGCTCGTACCGTGAGTAATAATGCGATTTAAATC
miR99a-5p	F	TCGAGATTTAAATCACAAGATCGGATCTACGGGTTGC
	R	GGCCGCAACCCGTAGATCCGATCTTGTGATTTAAATC
miR374a-5p	F	TCGAGATTTAAATCACTTATCAGGTTGTATTATAAGC
	R	GGCCGCTTATAATACAACCTGATAAGTGATTTAAATC
miR15b-5p	F	TCGAGATTTAAATTGTAAACCATGAUGTGCTGCTAGC
	R	GGCCGCTAGCAGCACATCATGGTTTACAATTTAAATC
miR27a-3p	F	TCGAGATTTAAATGCGGAACTTAGCCACTGTGAAGC
	R	GGCCGCTTCACAGTGGCTAAGTTCCGCATTTAAATC

Supplementary Table 6. Primer sequences

Non-genomic Reporter		Sequence 5'→3'
NG1-Psicheck2.0	F	TCGAGATTTAAATTCGATTCAGTCAGTCAAGCTGCGC
	R	GGCCGCGCAGCTTGACTGACTGAATCGAATTTAAATC
NG2-Psicheck2.0	F	TCGAGATTTAAAT GACCTGAAACTGGGACTTTGCGGC
	R	GGCCGCCGCAAAGTCCCAGTTTCAGGTCATTTAAATC
NG3-Psicheck2.0	F	TCGAGATTTAAATGGTGCAAATCGCTTTCCGGAAAGC
	R	GGCCGCTTTCCGGAAAGCGATTTGCACCATTTAAATC
NG4-Psicheck2.0	F	TCGAGATTTAAATTGGCGTAACTCCGCGTATGGGC
	R	GGCCGCCCATACGCGGAGTTACGCCAATTTAAATC

Mimics		Sequence 5'→3'
NG1-mimic	F	UCGAUUCAGUCAGUCAAGCUGC
	R	AGCUUGACUGACUGAAUCGAUU
NG2-mimic	F	GACCUGAAACUGGGACUUUGCG
	R	CAAAGUCCCAGUUUCAGGUCUU
NG3-mimic	F	GGUGCAAAUCGCUUUCCGGAAA
	R	UCCGGAAAGCGAUUUGCACCUU
NG4-mimic	F	UGGCGUAACUCCGCGUAUGG
	R	AUACGCGGAGUUACGCCAUU

Supplementary Table 6. Primer sequences
